# Exploring Internal Medicine by a Comparative Schematic Analysis of the Long-Term Outcomes of Anticoagulation Therapy in Atrial Fibrillation

**DOI:** 10.7759/cureus.82475

**Published:** 2025-04-17

**Authors:** Wajiha Gul, Sehrish Qaiser, Syed Muzaffar Abbas, Dipendra Singh, Mars Christian Aragon Sta Ines, Lareb Asad, Zala Aftab, Muhammad Hussain Shah, Ehsan Ul Haq Mzahri

**Affiliations:** 1 General Internal Medicine, Midland Metropolitan University Hospital, Birmingham, GBR; 2 Acute and General Medicine, University Hospitals Birmingham, Birmingham, GBR; 3 General Medicine, Bangor Hospital, Wales, GBR; 4 Orthopedics, Soo-Jung Hospital, Rajpur Doti, NPL; 5 Medical Education and Emergency Medicine, University Hospital Coventry, Coventry, GBR; 6 Pathology, Peoples University of Medical and Health Sciences, Nawabshah, PAK; 7 Internal Medicine, Medical Teaching Institute - Hayatabad Medical Complex, Peshawar, PAK; 8 Medicine, Khyber Girls Medical College, Peshawar, PAK; 9 Pathology, Dow University of Health Sciences, Dow International Medical College, Karachi, PAK; 10 Health Sciences and Pathology, University of the Punjab, Lahore, PAK; 11 Pathology and Oncology, Forman Christian College, Lahore, PAK; 12 Pathology, University of Indonesia, Jakarta, IDN

**Keywords:** anticoagulation, atrial fibrillation, internal medicine, outcomes, prevention, stroke, therapy

## Abstract

Atrial fibrillation (AF) is a common arrhythmia with increased risks of stroke and other cardiovascular complications. This research examined the long-term outcomes of anticoagulation therapy in AF patients, focusing on its benefits for stroke reduction, bleeding hazards, and survival rates. Researchers conducted an extensive literature search that combined PubMed, Scopus, Web of Science, and Google Scholar to retrieve publications from 2015 to 2025. The search focused on keywords related to anticoagulation therapy and its connection to atrial fibrillation, stroke prevention, as well as both long-term outcomes and bleeding risks. The data extraction process was performed by two independent reviewers, while the assessment of study quality relied on the Newcastle-Ottawa Scale and the Cochrane Risk of Bias Tool. The evaluation of evidence quality followed the GRADE approach. A total of 12 studies were included in this review after full screening. Direct oral anticoagulants (DOACs) exhibited either matching or superior stroke prevention performance compared to warfarin while showing lower major bleeding occurrence according to existing research findings. Studies demonstrated that patients receiving DOACs had longer survival rates when examining death rates. The review demonstrated that anticoagulation medication shows strong clinical results as a treatment strategy for prolonged atrial fibrillation cases. Further research must expand with prolonged follow-up examinations to establish data on the safety and effectiveness of DOACs compared to warfarin and to create better guidelines for the long-term anticoagulation treatment of AF patients.

## Introduction and background

Atrial fibrillation (AF) is a widespread cardiac arrhythmic condition, as its prevalence has been increasing among older individuals worldwide [1]. The medical complications of stroke, systemic embolism, and mortality are more frequent among patients with AF [2]. The most effective treatment for AF patients is anticoagulation therapy because it reduces their thromboembolic risks, although higher risk stroke levels determine treatment necessity [3]. Warfarin was formerly considered the primary anticoagulant therapy until direct oral anticoagulants (DOACs) emerged as alternatives offering stable dosages and diminished drug-drug and food-drug interactions [4]. Medical experts continue to debate the relationship between stroke reduction and bleeding risks and survival consequences that occur from anticoagulation procedures [5].

Research indicates that DOAC reduces bleeding events, but it raises unknown safety considerations for elderly patients and those with renal issues [6]. Healthcare professionals select between warfarin and DOACs for anticoagulation treatment by considering individual patient aspects such as multiple medical conditions and renal function, together with age-related elements [7,8].

This research examined how anticoagulation treatment for AF patients affects stroke prevention rates together with bleeding susceptibility, and hospital admissions together with survival patterns during long-term follow-up. This research built its objective from extended studies of internal medicine optimization practices with anticoagulation therapy in patients who have AF.

## Review

Methodology

The review method adopted the Preferred Reporting Items for Systematic Reviews and Meta-Analyses (PRISMA) guidelines 2020 to achieve a comprehensive evaluation of extended anticoagulation therapy results for AF patient populations. Studied publications satisfied research conditions for randomized controlled trials (RCTs) along with observational studies while collecting information from short and long-term anticoagulation treatment follow-ups. Research that examined anticoagulation therapy among AF patients who received anticoagulants and reported stroke prevention effects, provided bleeding risk assessments, as well as mortality data and hospital admission statistics, was eligible for inclusion.

A comprehensive research was performed on PubMed, Scopus, Web of Science, and Google Scholar databases for studies published from 2015 to 2025. The research utilized the keywords "atrial fibrillation", "anticoagulation therapy", "long-term outcomes", "stroke prevention", "DOACs", and "warfarin" for the search. Search results were enhanced by the use of Boolean operators, and the study included only English-language studies.

The two evaluators conducted independent quality checks on every study, beginning with the title and abstract phase, followed by full-text analysis. Disagreements were resolved by discussing any conflicting items with a third expert when needed. Two reviewers independently performed data extraction using a standardized form for collecting essential study information involving patient characteristics, therapy use, and clinical outcomes such as stroke occurrences, bleeding events, and mortality rates. The study quality assessment tool for observational research was the Newcastle-Ottawa Scale, while the Cochrane Risk of Bias Tool was used for RCTs.

The review compared warfarin to DOACs according to various clinical measures (stroke prevention, bleeding complications, and mortality). The variability among the studies did not allow for a combined statistical analysis. The study's evidence quality assessment adopted the GRADE approach for integrating the quality of the findings with study design, risk of bias, and consistency of research results. The research design provided a reliable method to evaluate long-term outcomes of anticoagulation therapy in AF patients.

Results

The long-term outcomes of AF anticoagulation therapy were evaluated in 12 studies that analyzed stroke prevention, bleeding hazards, and patient mortality statistics. The research literature was extensively reviewed using databases from 2015 to 2025 for study selection.

**Figure 1:**
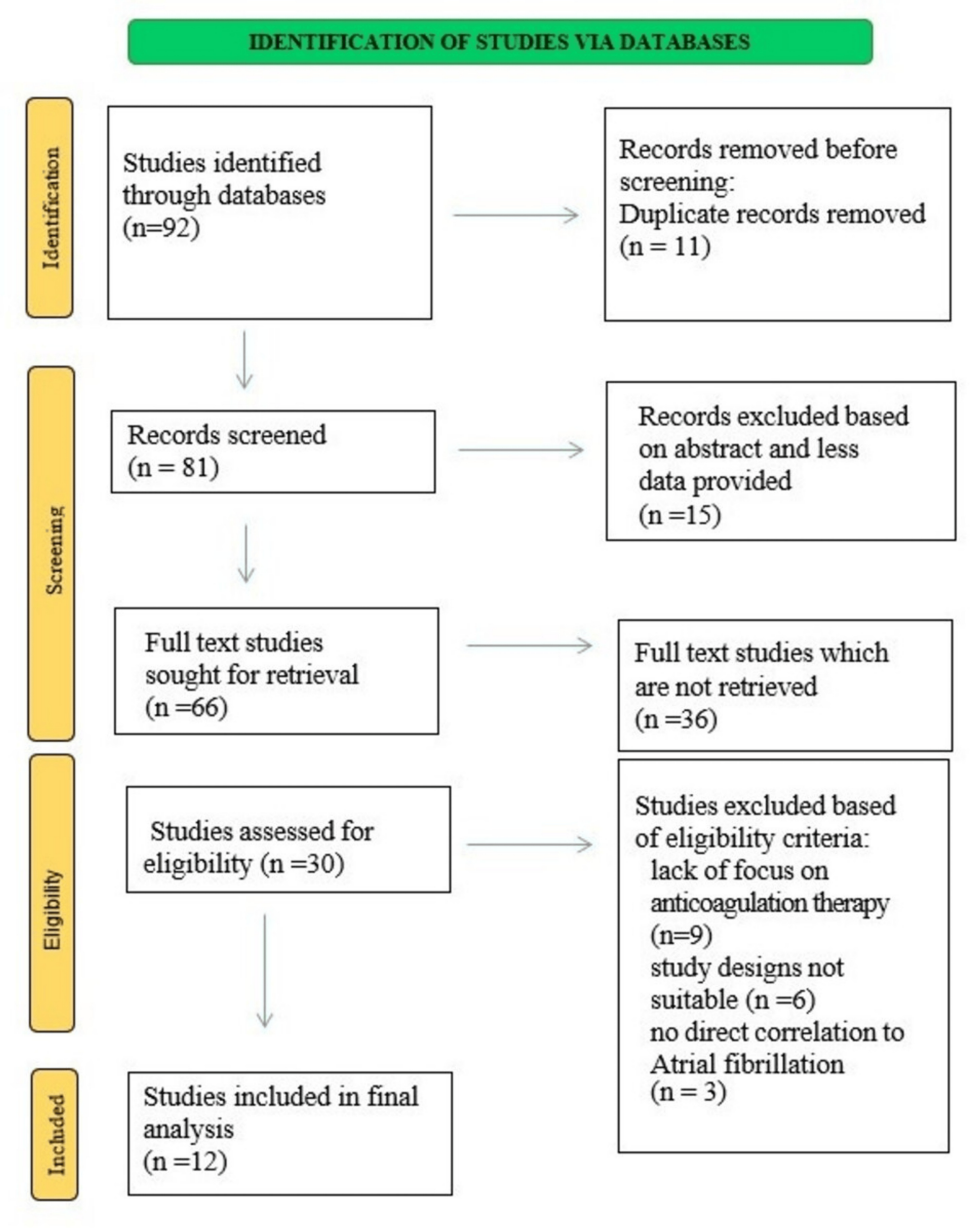
PRISMA flow diagram PRISMA: Preferred Reporting Items for Systematic Reviews and Meta-Analyses

A total of eight observational cohort studies, three retrospective cohort studies, and one network meta-analysis were included among the selected studies. Each study included between 106 and 171,700 participants, with a median of 59,172 individuals throughout the research period. Atrial fibrillation patients made up the majority of the study subjects, including those receiving direct oral anticoagulants (DOACs) and warfarin treatment. Figure 1 demonstrates the steps of the study selection process. 

Reviews of studies documented that DOACs (apixaban, rivaroxaban, dabigatran, and edoxaban) were associated with reduced risks of stroke, systemic embolism, and bleeding complications relative to the older anticoagulant warfarin, while also demonstrating beneficial effects for new anticoagulant medications. The use of warfarin was associated with an increased risk of major bleeding and stroke among patients. The biomarkers from these treatments could serve as essential markers to assess treatment success and ensure patient safety, according to the findings of Ando et al. (2025), Dawwas et al. 2025, and Lan et al. (2025) [9, 15, 20]. A summary of individual findings of the selected studies can be seen in Table 1.

**Table 1:** Systematic review table showcasing characteristics and key findings of individual studies AF: Atrial Fibrillation; DOAC: Direct Oral Anticoagulant; EVT: Endovascular Treatment; HR: Hazard Ratio; IS: Ischemic Stroke; NOACs: Novel Oral Anticoagulants; NVAF: Non-Valvular Atrial Fibrillation; OR: Odds Ratio; SE: Systemic Embolism; VKA: Vitamin K Antagonist.

Study	Study Design	Sample Size	Anticoagulation Therapy	Key Findings
Ando T et al. (2025) [9]	Observational cohort study	29,142	DOACs (Direct Oral Anticoagulants), Warfarin	DOACs were associated with lower risks of bleeding, mortality, and stroke
Imaoka Y et al. (2024) [10]	Retrospective, multi-center study	6,984 patients with atrial fibrillation (AF) who received EVT between 2018 and 2020.	Warfarin, DOACs	Prior DOAC use may boost EVT outcomes in high-risk AF patients.
Carnicelli AP et al. (2022) [11]	Network Meta-Analysis	71,683 patients	Standard-dose DOAC, Lower-dose DOAC, Warfarin	Standard-dose DOACs: Better for stroke and bleeding vs. Warfarin, Safer and more effective than warfarin.
Fralick M et al. (2020) [12]	Retrospective Cohort Study	59,172 on apixaban, 40,706 on rivaroxaban	Apixaban, rivaroxaban	Apixaban was associated with a lower incidence of ischemic stroke/systemic embolism
Veltkamp R et al. (2025) [13]	Multicenter, open-label, randomized Phase 3 Trial	319 participants (158 DOAC group, 161 no anticoagulant group)	DOACs (Direct Oral Anticoagulants) vs. No anticoagulation	DOACs significantly reduced the risk of ischemic stroke
Atreja N et al. (2025) [14]	Retrospective cohort study	171,700 patients with NVAF who switched from warfarin to DOACs	Apixaban, rivaroxaban, dabigatran	Apixaban: Lower stroke/SE and major bleeding rates vs. dabigatran and rivaroxaban
Dawwas GK et al. (2025) [15]	Retrospective cohort study	81667	Apixaban, dabigatran, rivaroxaban, edoxaban, warfarin	DOACs: Lower stroke/embolism and bleeding vs. warfarin. Apixaban & rivaroxaban: Most reduction in stroke/bleeding.
Vicario T et al. (2025) [16]	Retrospective study	106 atrial fibrillation (AF) patients with ischemic stroke (IS)	Direct Oral Anticoagulants (DOACs) vs Vitamin K Antagonists (VKA)	DOACs: Lower risk of severe stroke and reduced mortality vs. Warfarin.
Rahme E et al. (2021) [17]	Retrospective cohort study	10,893 patients on apixaban, 10,190 on rivaroxaban, 5,884 on dabigatran	DOACs – apixaban, rivaroxaban, dabigatran vs warfarin	Standard-dose DOACs: better bleeding (apixaban HR 0.63, dabigatran HR 0.47). Death risk: Lower for all DOACs.
Gupta K et al. (2019) [18]	Retrospective cohort study using US Department of Defense (DOD) data	41,001 patients	Direct oral anticoagulants (DOACs) – apixaban, dabigatran, rivaroxaban vs warfarin	Apixaban: Lower stroke/SE (HR 0.55) and major bleeding (HR 0.65) vs. warfarin. Dabigatran & rivaroxaban: Similar stroke/SE and bleeding risks to warfarin
Grymonprez M et al. (2023) [19]	Retrospective cohort study using Belgian nationwide data (2013–2019)	254,478 AF patients	NOACs vs VKAs (dabigatran, apixaban, rivaroxaban, edoxaban)	NOACs: Lower stroke/SE, mortality, bleeding vs. VKAs. Apixaban: Best safety, followed by dabigatran.
Lan Y et al. (2025) [20]	Retrospective multicenter cohort study	Retrospective Multicenter Cohort Study	NOACs (dabigatran, rivaroxaban, apixaban, or edoxaban) vs. Warfarin	NOACs: Lower minor bleeding (OR 0.70) and mortality (OR 0.57) vs. Warfarin, in females: Increased major bleeding risk (OR 2.28).

This review synthesizes existing research on the effectiveness and safety of anticoagulant treatments and compares DOACs with warfarin by analyzing different patient groups. Patients undergoing transcatheter aortic valve replacement (TAVR) experienced reduced bleeding risks, mortality, and stroke events with DOACs compared to warfarin, according to the study by Ando et al. (2025) [9]. Previous DOAC treatment of atrial fibrillation patients undergoing endovascular therapy led to improved healthcare results for AF patients with high-risk profiles, according to Imaoka et al. (2024) [10]. Standard-dose treatment with DOACs proved to offer better protection against stroke and bleeding events in comparison to warfarin, according to Carnicelli et al.'s (2022) network meta-analysis [11]. On the basis of their research, Fralick et al. (2020) demonstrated that apixaban surpassed rivaroxaban regarding stroke/systemic embolism occurrences and bleeding events [12].

The randomized multicenter trial conducted by Veltkamp et al. (2025) established that DOACs successfully reduced the risk of ischemic stroke better than no anticoagulation among ischemic stroke patients [13]. Patients moving from warfarin to DOACs experienced better outcomes regarding stroke/systemic embolism prevention, together with major bleeding prevention, when using apixaban compared to dabigatran and rivaroxaban, according to Atreja et al. (2025) [14]. A study conducted by Dawwas et al. (2025) showed that apixaban and rivaroxaban performed best in stroke/embolism prevention and bleeding prevention among a large patient population compared to warfarin therapy [15]. Results from Vicario et al.'s (2025) study showed that DOACs reduced both stroke mortality rates and limited the severity of strokes better than warfarin in patients with ischemic stroke [16]. Rahme et al. (2021) showed that standard-dose DOACs decrease mortality and bleeding risks more than warfarin in people needing anticoagulation therapy [17].

However, rivaroxaban and dabigatran proved comparable to warfarin in risk outcomes [18, 19]. Lan et al. (2025) analyzed novel oral anticoagulants (NOACs) and established that these medications produced reduced rates of mortality and minor bleeding risks than warfarin, but females experienced elevated major bleeding tendencies [20].

The randomized trials managed study conditions well, but researchers lacked complete data regarding outcomes while still facing blinding challenges. Disputes between reviewers needed third-party guidance in addition to their debates for resolution. See Table 2 and Table 3. The GRADE assessment system is a systematic method that rates the robustness of evidence in the reviewed studies. According to research findings, the safety and effectiveness profiles of DOACs exceeded those of warfarin, despite various study weaknesses, including limited participant numbers. The encouraging evidence about DOACs requires additional, extensive multi-site trials to develop robust clinical usage recommendations.

**Table 2:** Risk of bias assessment of individual RCTs "+" indicates a low risk of bias, "±" indicates an unclear or moderate risk of bias, and "-" indicates a high risk of bias.

Study	Sequence Generation – Selection Bias	Allocation Sequence Concealment – Selection Bias	Blinding Of Participants and Personnel – Performance Bias	Blinding Of Outcome Assessment – Detection Bias	Incomplete Outcome Data	Selective Outcome Reporting	Other Bias
Veltkamp R et al., 2025 [13]	+	+	±	+	±	+	±

**Table 3:** Risk of bias assessment of individual observational studies

Study	Selection (max 4)	Comparability (max 2)	Outcome (max 3)	Total Score (max 9)
Ando T et al., 2025 [9]	★★★★	★★	★★★	9
Imaoka Y et al., 2024 [10]	★★★★	★	★★★	7
Carnicelli AP et al., 2022 [11]	★★★★	★★	★★★	9
Fralick M et al., 2020 [12]	★★★★	★	★★★	8
Atreja N et al., 2025 [14]	★★★★	★	★★★	8
Dawwas GK et al., 2025 [15]	★★★★	★	★★★	8
Vicario T et al., 2025 [16]	★★★★	★	★★★	8
Rahme E et al., 2021 [17]	★★★★	★	★★★	8
Gupta K et al., 2019 [18]	★★★★	★	★★★	8
Grymonprez M et al., 2023 [19]	★★★★	★	★★★	8
Lan Y et al., 2025 [20]	★★★★	★	★★★	8

Discussion

Multiple studies demonstrated that the findings of this systematic review align with growing evidence supporting the use of DOACs instead of warfarin for atrial fibrillation patients undergoing long-term anticoagulation treatment. Numerous studies from this review, along with prior meta-analyses, established that DOACs provide superior stroke prevention and systemic embolism outcomes while reducing bleeding hazards. The results are consistent with those from the RE-LY (dabigatran), ROCKET-AF (rivaroxaban), and ARISTOTLE (apixaban) trials, which demonstrate that DOACs present efficacy or better performance and equivalent or better safety versus warfarin [21,22].

A variety of research has shown that stroke prevention benefits and reduced bleeding incidents are common outcomes for patients taking DOACs. Apixaban demonstrates superior safety characteristics compared to warfarin. Another study confirmed that none of the NOACs, including apixaban, resulted in major bleeding or mortality at rates more than vitamin K antagonists (VKAs). The findings from analyses showed that, despite their superior advantages in safety, DOACs lead to bleeding problems that are specific to elderly patients among other subgroups [23,24]. Previous researchers support this concern because they highlight the significance of proper bleeding risk control for delicate patient groups [25,26]. Study findings become less reliable since researchers have employed diverse study designs and have conducted short-term follow-ups in some research. The analysis results might have been favorably skewed toward significant outcomes because of publication biases. Multiple additional research fields require further development to expand current knowledge [27].

Randomized controlled trials with extended follow-up need to be executed with diverse participant groups encompassing elderly people and patients who have multiple health conditions to measure DOACs' long-term performance and safety. The determination of DOAC cost-effectiveness in resource-limited settings requires more scientific investigation. Scientists need to research new treatment methods alongside biomarker-based individualized therapy approaches to harmonize security and economical treatment choices for patients. This effort will facilitate the development of precise anticoagulant care.

## Conclusions

This systematic review corroborates the mounting evidence that demonstrates that DOACs should be the preferred therapy for preventing strokes in atrial fibrillation patients over the long term. DOACs prove to be better than warfarin regarding safety and efficacy but require proper attention before prescribing to at-risk patient groups.

The findings require focused attention from clinical practitioners, policy makers, and researchers in order to better define treatment protocols while increasing therapy availability and solving unknown aspects via quality-focused scientific work. The advancement of new treatment approaches with biomarker-based individualized therapy needs careful research by scientists to offer patients safe and affordable healthcare options. The effort will contribute to developing precise anticoagulant mentoring.
